# First report of *Besnoitia bennetti* in Irish donkeys: an emerging parasitic disease in Europe

**DOI:** 10.1186/s13620-024-00263-2

**Published:** 2024-02-14

**Authors:** Stacy H. Tinkler, Luca Villa, Maria Teresa Manfredi, Nicola Walshe, Hanne Jahns

**Affiliations:** 1 Veterinary Department, The Donkey Sanctuary Ireland, Hannigan’s Farm, Liscarroll, Mallow Co. Cork Ireland; 2 Department of Veterinary Medicine and Animal Sciences, Università Degli Studi Di Milano, Via Dell’Università, 6, 26900 Lodi, Italy; 3 Pathobiology Section, School of Veterinary Medicine, University College Dublin, Belfield, Dublin, D04W6F6 Ireland

**Keywords:** Equus asinus, Parasite, Protozoa, Dermatitis, Emerging disease, Scleral cysts, Sarcoid

## Abstract

**Background:**

This is the first report of *Besnoitia bennetti* in donkeys in Ireland. *B. bennetti*, an apicomplexan protozoan parasite specific to equids, is an emerging pathogen in Europe. This parasite forms chronic intracytoplasmic cysts in cells of the mesenchymal lineage, mainly fibroblasts, in the skin, sclera and mucosa. Clinical signs in affected equine hosts vary from mild to severe debilitating disease. Little is known of the phylogeny, epidemiology or transmission of *B. bennetti* infection in donkeys, mules or horses.

**Case presentation:**

Two cases of besnoitiosis in donkeys are presented. Both donkeys were born and raised in theSouthwest of Ireland. The first case was a 2.5-year-old donkey that had a suspect sarcoid removed, while the second case,a 2-year-old donkey, had a biopsy of nodular dermatitis of the muzzle. Diagnosis was made by histopathology and the parasite species, *B. bennetti*, was confirmed by PCR followed by sequencing and microsatellite analysis. Both donkeys had high antibody titres against *Besnoitia* spp. Small (0.5 mm) scleral, conjunctival and dermal cysts over the muzzle were subsequently observed in both animals. Treatment with trimethoprim sulfadiazine for 30 days did not lead to clinical resolution. The findings are compared to the cases of besnoitiosis in donkeys reported in the past 10 years throughout Europe.

**Conclusions:**

Besnoitiosis should be considered as a differential diagnosis for chronic skin disease particularly in cases of cutaneous masses, non-pruritic dermatitis, and dermatitis that is not responsive to treatment in donkeys and other equids. Future studies are needed to investigate the prevalence of the disease in Irish donkeys, the spread of the disease and the potential impact on the health and welfare of the donkeys.

**Supplementary Information:**

The online version contains supplementary material available at 10.1186/s13620-024-00263-2.

## Background

Besnoitiosis is a parasitic disease affecting wild and domestic animals world-wide [1]. It is caused by *Besnoitia* spp., a cyst forming coccidia (phylum Apicomplexa, class Conoidasida, order Eucoccidiorida, family Sarcocystidae), closely related to other pathogenic protozoa of domestic and wild animals, e.g., *Toxoplasma gondii*, *Neospora* spp*.* and *Sarcocystis* spp. [2]. Among 10 recognized species within the genus *Besnoitia* are four closely related species infecting domestic and wild ungulates; *B. besnoiti* (cattle), *B. bennetti* (equids)*, B. caprae* (goats), and *B. tarandi* (cervids) [3, 4]*. Besnoitia* spp. has a two-host life cycle using prey as the intermediate host and a predator as the definitive host [1, 5]. Sexual reproduction occurs in the intestine of the definitive host in which unsporulated oocysts are produced and shed in the faeces. They then sporulate in the environment. When ingested by a susceptible intermediate host, the sporulated oocysts produce tachyzoites, which circulate in the blood stream [1, 6] during the acute phase of disease. During the chronic phase tachyzoites migrate into the connective tissue mainly in the dermis, sclera and mucosa where they form large tissue cysts, filled with bradyzoites, in cells of the mesenchymal lineage, mainly fibroblasts [7, 8]. Despite the designation of *B. besnoiti* and *B. bennetti* as emerging pathogens [9, 10], no definitive host in which sexual development can take place has been identified for either species [7]. Therefore, the life cycle, and mode of transmission of *Besnoitia* spp. in equids and ruminants remains unknown [1, 11]. In cattle, possible direct transmission via licking and natural mating and iatrogenic transmission during prophylactic and treatment procedures within the herd [3, 12] have been postulated. Further, mechanical transmission by hematophagous insects (*Glossina*, *Stomoxys* and tabanids) has been demonstrated in cattle [13, 14].

**Fig. 1:**
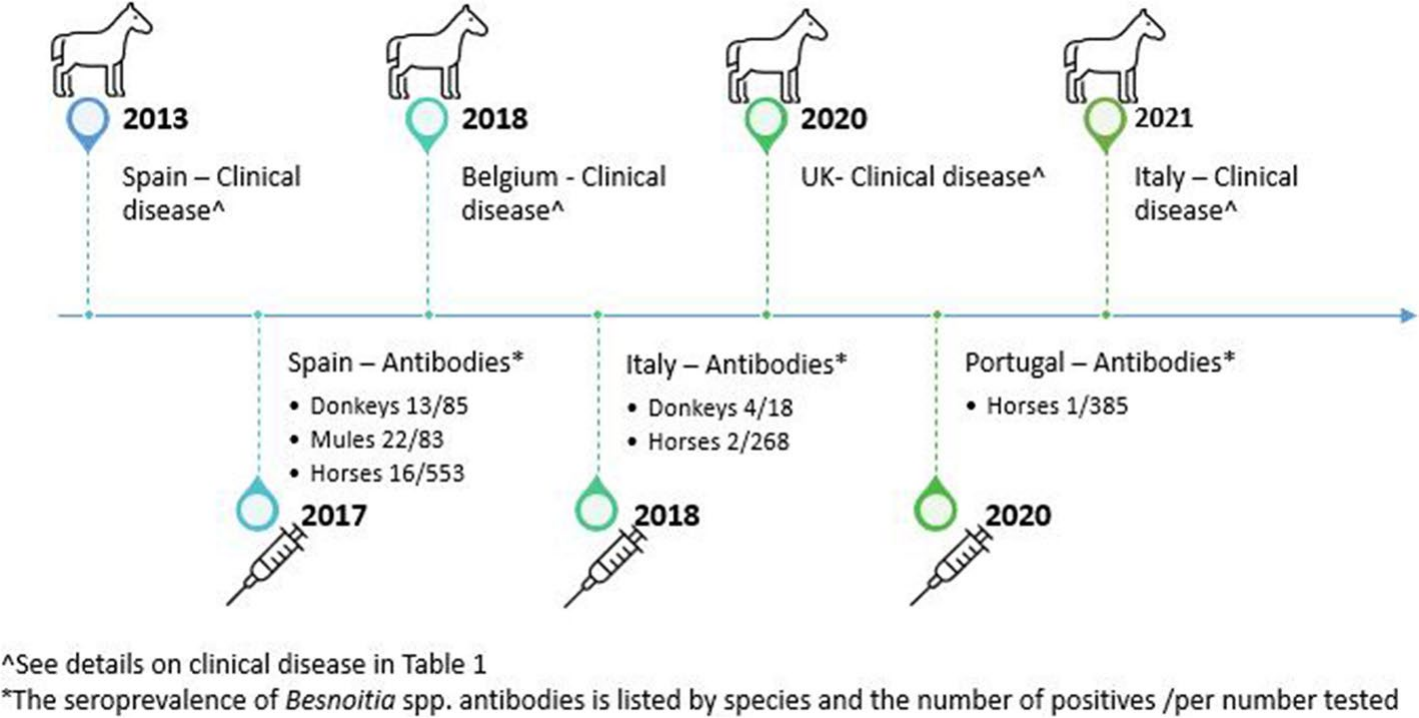
The diagram shows the recent emergence of *Besnoitia* spp. infection in equids in Europe, by date of reporting, since its first description in a French horse in 1922

Reports of besnoitiosis in equids date back to 1927 in Africa [15–18]. Outside of Africa, it has been reported in the United States since 1973 [19], where it is considered an emerging disease of donkeys [1, 5, 20, 21]. In Europe, the first clinical case was described in a horse in France by Henry and Masson in 1922 (cited by Pols 1960 [22]), and since 2013 sporadic cases of besnoitiosis in donkeys and seroprevalence of *Besnoitia* spp antibody. in equids have been reported in several European countries (Fig. 1). Of these European cases, *B. bennetti* has only been confirmed in one British donkey [23] and one Belgian donkey [24] by molecular methods.

Besnoitiosis in equids is usually a non-fatal disease, characterized primarily by multi-focal white miliary nodular lesions (parasitic cysts) in the skin in a variety of sites: over the face and body, within the nares, on the internal and external pinnae, vulva, perineum and on the legs [1, 5, 25]. The most common and unique feature is the presence of scleral and conjunctival parasitic cysts, often referred to in the literature as ‘scleral pearls’ [1, 5, 21, 23–28]. Cysts have also been observed on the laryngeal mucosa [21, 23, 27], rarely in lamellae and laminar chorium [23], oral mucosa [21, 24] and pharyngeal and tracheal mucosa [27] (Table 1). Rarely, more severe disease, seen as weight loss, ventral oedema and dyspnoea, has been reported in equine species [1, 5, 18, 21, 27].

**Table 1:** Signalment, clinical signs and diagnoses of cases of besnoitiosis in European donkeys reported since 2013

**Country**	**Year of reporting**	**No. of Cases**	**Age range**	**Clinical signs**					**Diagnostics**		
				**Scleral & conjunctival cysts**	**Skin Lesions (distribution)**	**Mucosal lesions (distribution)**	**Other**	**Haematology Biochemistry**	**Histopathology**	**Serology**	**Molecular**
**Spain** (Zafra et al. 2013) [28]	2013	7	1-7yo	No	Nose, ears, shoulders perineum	Lower lip	No	NA	Yes	NA	NA
**Belgium** (Lienard et al. 2018) [24]	2018	2	2yo	Yes	Muzzle, flank, neck	No	Poor BCS, Pruritis, Anorexic	Anaemia eosinophilia, increase gamma globulin fraction	Yes	WB	qPCR, ITS-1 seq **Confirmed** ***B. Bennetti***
			10yo	yes	Nares	Labial	No	Eosinophilia	No	WB	qPCR negative
**United Kingdom** (Elsheikha et al. 2020) [23]	2020	8	5-20yo	Yes (1 case)	Face, abdomen, penile sheath	Lip	Corneal ulcer, weight loss, Conjunctivitis sarcoids	NA	Yes	IFAT, Immuno blot (1 case)	PCR, microsatellite typing (1 case), **Confirmed** ***B. Bennetti***
		12 (postmortem)	5-32yo	Yes	Eyelid, Muzzle	Larynx	Conjunctivitis laminae, Sarcoids	NA	Yes	Immuno blot (4 cases)	NA
**Italy** (Villa et al. 2021) [25]	2021	2	1yo	Yes	Neck, hindlimbs pinnae	No	Poor BCS, dull, rough hair coat	Mild anaemia, hypoalbuminaemia with decreased albumin/globulin ratio, eosinophilia, lymphocytosis, elevated ALP	No	WB	PCR, ITS-1 seq Not confirmed *B. Bennetti*
**Ireland** (this study)	2023	2	2yo	Yes	Muzzle, nares	No	No	WNL	Yes	ELISA, IFAT, WB	PCR, ITS-1 seq, microsatellite typing **Confirmed** ***B. Bennetti***
			2.5yo	Yes	Muzzle	No	sarcoid	WNL	Yes	ELISA, IFAT, WB	PCR, ITS-1 seq, microsatellite typing **Confirmed** ***B. Bennetti***

*NA* Not applicable, *WNL* Within normal limits, *seq* Sequencing

This study describes for the first time the clinical presentation, diagnosis and case management of *B. bennetti* infection in two donkeys in Ireland*.* The findings are compared to cases of besnoitiosis in donkeys reported in Europe over the past ten years and are discussed.

## Case presentation

### Case 1

A 2.5-year-old donkey jenny was presented for assessment of a dermal mass on her muzzle (6.5 mm × 7.7 mm) that was suspected to be a fibroblastic sarcoid (Fig. 2b) in May 2022. The mass was associated with a scar that she had sustained as a foal from the bite by another donkey. The jenny was otherwise clinically healthy, weighed 211 kg, and was in good body condition. She was born on the farm (The Donkey Sanctuary Ireland, Mallow, Co. Cork) and was housed in a group of 76 donkeys, mixed males, and females, between 2 and 10 years of age, which were on a diet of ad libitum straw with limited haylage in autumn/winter and limited grass pasture and ad libitum straw in spring/summer. They were routinely vaccinated for equine influenza and tetanus, routinely monitored both for endo (liver flukes, strongyles, lungworms, ascarids) and ectoparasites and treated as needed. The donkeys were weighed monthly, and condition scored to monitor for any unusual weight gain or loss in which case they would have been identified for clinical examination and/or any necessary and appropriate diagnostic tests (haematology, biochemistry, faecal anaylsis etc.).

**Fig. 2:**
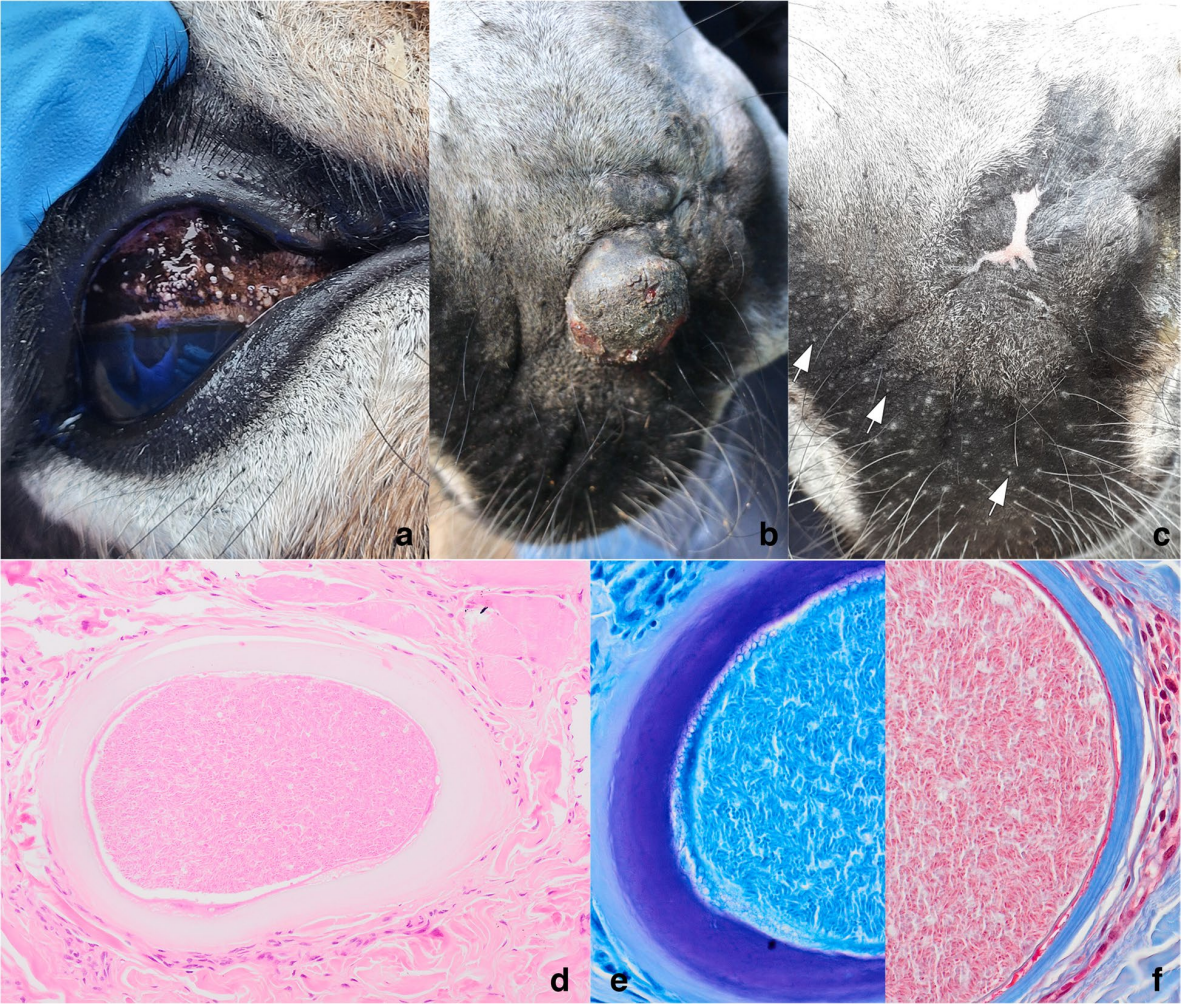
Case 1, donkey, sarcoid and *Besnoitia* cysts: **a** numerous white pinpoint cysts present on the conjunctiva, **b** Initial presentation of raised broad based ulcerated dermal nodule on the muzzle, **c** Muzzle four weeks after surgery and trimethoprim sulfadiazine treatment, pinpoint white cysts visible (arrows), **d** Photomicrograph illustrating a tissue cyst with the characteristic three layers: a hyaline outer layer (Giemsa positive (**e**) consisting of collagen (Masson trichrome positive (**f**)), a middle layer composed of host cell cytoplasm with multiple nuclei and a thin inner membrane enclosing a parasitophorous vacuole with the bradyzoities, haematoxylin eosin

She was deemed fit for surgical removal of the mass via laser after initial clinical exam, and routine pre-anaesthetic blood tests (haematology and biochemistry) were all found to be within normal limits.

The jenny was pre-medicated with a combination of intravenous flunixin meglumine, 1.1 mg/kg (Finadyne, Intervet Animal Health, Dublin, Ireland), acepromazine, 0.03 mg/kg (Acecare, Duggan Vet Supplies, Thurles, Ireland), romifidine, 0.15 mg/kg (Sedivet, Boehringer Ingelheim, Vetmedica GmbH, Rhein, Germany) and butorphanol, 0.02 mg/kg (Torbugesic, Zoetis UK Limited, UK). Anaesthesia was induced with ketamine, 2.8 mg/kg (Narketan, Vetoquinol Ire, Dublin, Ireland) and diazepam, 0.1 mg/kg (Hameln Pharma Ltd, Gloucester, UK). Anaesthesia and surgery were both uneventful and the mass was removed, fixed in 10% buffered formalin, and submitted to the University College Dublin (UCD) Pathology Department for histopathological examination.

#### Histopathology

The sections of haired skin examined consisted mainly of a dermal sarcoid with narrow complete margins. Multifocally, within the periphery of the mass and in the adjacent superficial and deep dermis were several protozoal tissue cysts (*n* = 31 in 1cm^2^) extending to the margin of excision (Fig. 2d). The cysts were mainly oval and located in the cytoplasm of fibroblasts. Measurements were taken at the longest and at the widest position. The cysts measured on average 315 (172 to 455) µm in length and 247 (140 to 344) µm in width. The cyst consisted of an outer wall which was pale eosinophilic hyalinized and on average 19.4 (12 to 32) µm thick and stained blue with Masson trichrome (collagen) (Fig. 2f) and dark purple with Giemsa (Fig. 2e). This outer layer and the middle layer composed of the host cell were often slightly (artificially) separated with a smudged outline. The host cell had 3-6 elongated thin nuclei with fine stippled chromatin and rare intranuclear vacuoles (cytosequestrum). The eosinophilic cytoplasm formed a rim of about 2 µm around the parasitophorous vacuole. This vacuole contained large numbers of densely packed, crescent-shaped, eosinophilic, 3–5 µm bradyzoites with a small round basophilic nucleus (*Besnoitia* spp). All cysts were surrounded by layers of inflammatory cells, which ranged from 1 to 23 cells in thickness. These were composed of mainly lymphocytes, macrophages and few eosinophils. There were some degenerate cysts consisting of remnants of the pale eosinophilic outer wall, which was shrunken, infiltrated and occasionally obliterated by macrophages, lymphocytes, and eosinophils.

#### Post-operative care and follow-up

The surgical site was treated with 50:50 mix of 5-fluorouracil (Efudix 5%, Mylan IRE Healthcare Ltd, Dublin, Ireland) and Intra-site gel (Smith & Nephew Medical Ltd, Hull, England) twice daily for 10 days, and then pulse therapy for an additional 5 days on, 5 days off and 5 days on again for any remaining sarcoid tissue. Further examination of the donkey upon receiving the histologic diagnosis of besnoitiosis revealed numerous scleral and conjunctival cysts in both eyes (Fig. 2a), and the presence of additional cysts (0.5 -1 mm) on the muzzle. Treatment with 30 mg/kg trimethoprim sulfadiazine (Equibactin, Dechra, Oudewater, Netherlands) PO BID was initiated for 30 days (Derek Knottenbelt, personal communication) and photos were taken at 2-week intervals to monitor for any improvement in dermatitis, muzzle and ocular cysts. While the muzzle responded well to the sarcoid treatment and the surgical wound healed well (Fig. 2c), no significant clinical improvement regarding the presence of muzzle, conjunctival and scleral cysts was seen.

### Case 2

**Fig. 3:**
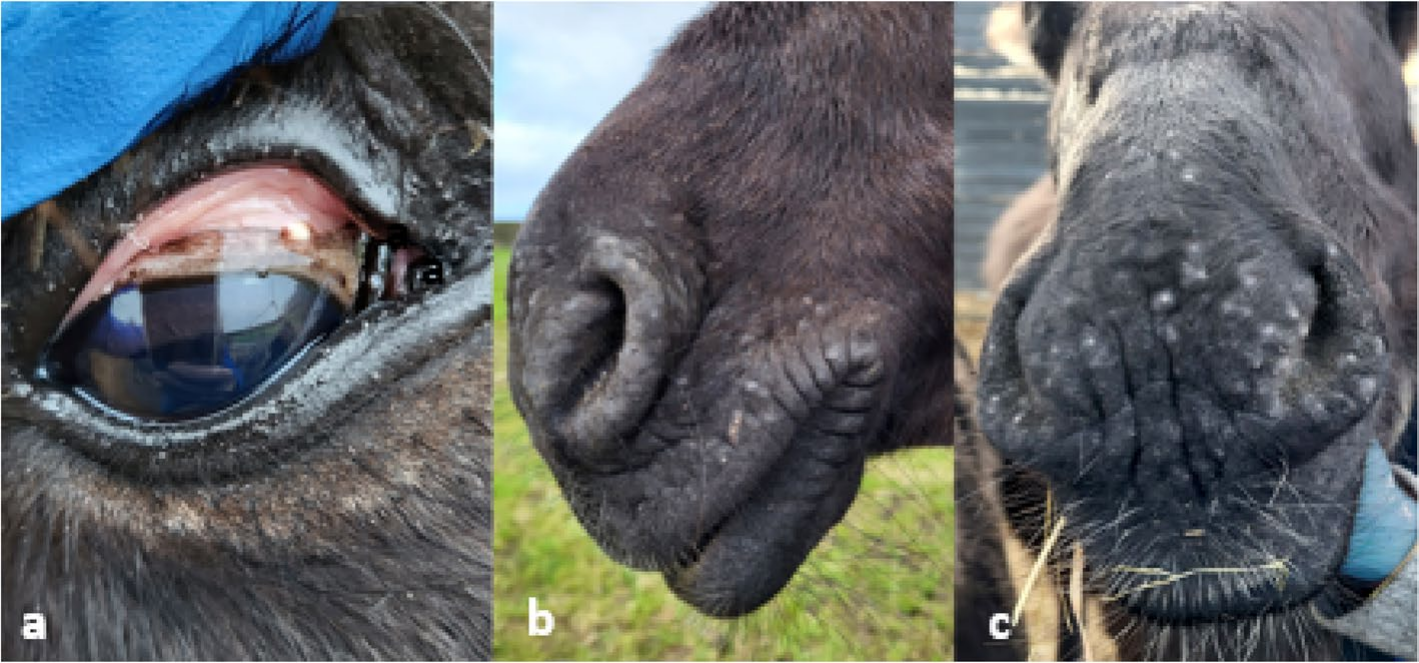
Case 2, donkey, dermatitis and *Besnoitia* spp. cysts: **a** few small scleral *Besnoitia* spp. cysts, **b** at initial presentation, multifocal to coalescing small raised often alopecic dermal nodules over the muzzle and nares, **c** the small raised white multifocal nodules were still present on the muzzle after 30- days of treatment

A second donkey, a 2-year-old gelding, from the same farm was presented for dermatitis on the muzzle, in and around the nares and commissures of the lips in late August 2022 (Fig. 3b). He was born on the farm and had been housed in a group of 10 donkeys, mixed males and females, ages 2—5 years old whose management was the same as described for case 1. Initial examination showed numerous focal areas of mild lichenification, alopecia and a few small white nodules of varying size (0.5–1.5 mm) were noted upon initial examination. The clinical exam parameters were within normal limits, with a temperature of 37.6 °C (36.5–37.8 °C reference range (RR)), heart rate of 56 beats per minute (bpm) (36–52 bpm RR), mild tachycardia being ascribed to stress, and a respiratory rate of 28 bpm (12–28 breaths per minute RR). He was in an ideal body condition with a condition score of 2.5/5, body weight 173 kg.

One month following the initial presentation many more small nodules with white, caseous material exuding from them were noted on the muzzle, nares and bridge of the nose of the gelding. A few more clusters of papules, some with crusts, were seen in the axilla and no pruritus was observed. Scleral and conjunctival cysts of varying size (0.5–2.5 mm) were found in and around both eyes (Fig. 3a) with no signs of conjunctivitis or any ocular discomfort. The donkey was sedated with a combination of detomidine (Domidine, Dechra, Eirovet, Netherlands) and butorphanol (Torbugesic, Zoetis UK Limited, UK) for a skin biopsy and blood was collected for hematology and biochemistry analysis. Flunixin meglumine, 1.1 mg/kg (Finadyne, Intervet Animal Health, Dublin, Ireland), was also given intravenously at time of sedation and biopsy. Subcutaneous blebs of 0.5 ml lidocaine hydrochloride (Lidobel, Belapharm, GmbH & Co, Vechta, Germany) were placed at each site. Six skin biopsies (4 × 6 mm punch, and 2 × 4 mm punch) were collected from the muzzle, nares, and the bridge of the nose. These were fixed in 10% buffered formalin and sent for histopathological examination. Haematology and biochemistry were unremarkable except for mild hypoglobulinemia (globulin 31 (32–48 g/dL RR).

#### Histopathology

**Fig. 4:**
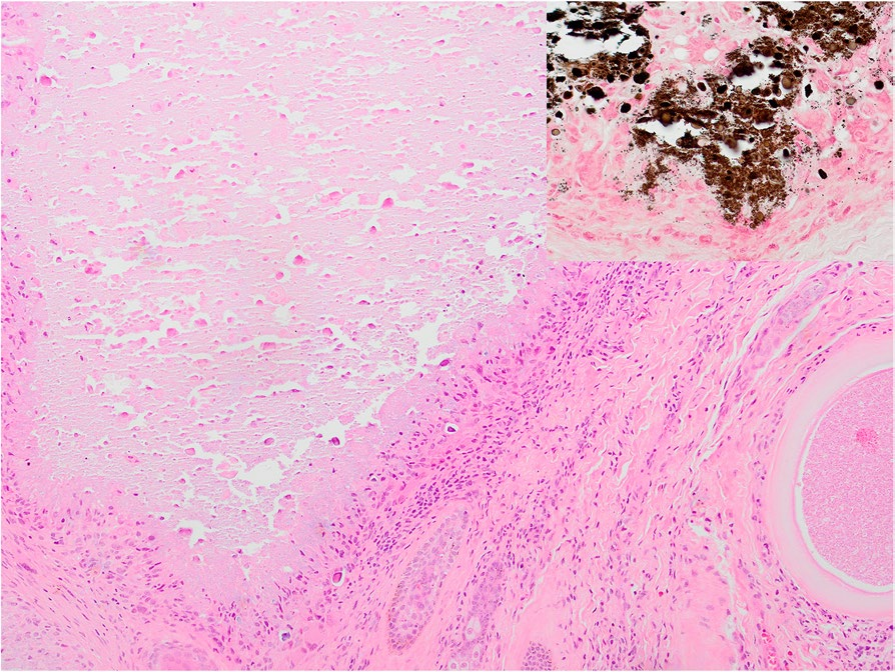
Case 2, donkey, dermis: representative photomicrograph of an area of basophilic material (calcified, van Kossa inset) surrounded by macrophages, few lymphocytes and fibroblasts (calcinosis circumscripta) and a typical *Besnoitia* sp. cyst in the adjacent dermis, haematoxylin eosin

In each skin biopsy, embedded into the collagen of the superficial and deep dermis were 3 to 4 oval protozoal (*Besnoitia* spp.) cysts. The cysts were on average 410 (274 to 562) µm long and 314 (155 to 449) µm wide. While the outer wall of the cysts, on average 28.6 (18 to 40) µm, was thicker than those observed in case 1, the morphological features of the host cell and the parasitophorous vacuole were the same. Pericystic inflammation was frequently present and ranged from none up to 10 layers of cells. The inflammatory infiltrate was composed of lymphocytes, macrophages, and few scattered eosinophils. Throughout the sections degenerate cysts were seen. These consisted of remnant outer walls and frequently showed bright eosinophilic cellular debris with pyknotic nuclei and basophilic brittle material (calcification) in the center. Large macrophages were observed palisading around the debris admixed with few lymphocytes and eosinophils. These granulomas were often surrounded by layers of fibroblasts and were on average 538 (232 to 858) µm long and 382 (178 to 436) µm wide. In addition, 5 large irregular foci of basophilic granular and brittle fragmented material replacing the collagen were seen in the dermis ranging between 0.76 and 4 mm in length and 0.68 to 12.6 mm in width (Fig. 4). These granular lakes also contained mineral deposits (van Kossa positive, Fig. 4 inset) and degenerate macrophages and were surrounded by a thin rim of macrophages, rarely multinucleated giant cells, that sometimes contained basophilic brittle material in the cytoplasm. The periphery was infiltrated by few lymphocytes and eosinophils and several layers of fibroblasts surrounded the granulomas. A diagnosis of dystrophic calcification (calcinosis circumscripta) was made in addition to chronic dermatitis with intradermal *Besnoitia* spp. cysts. There was also mild multifocal perivascular infiltration of plasma cells and eosinophils in the dermis.

Treatment with 30 mg/kg trimethoprim sulfadiazine (Equibactin, Dechra, Oudewater, Netherlands) PO BID was initiated for 30 days (Dr. Derek Knottenbelt, personal communication) and photos were taken at 2-week intervals to monitor for any improvement in dermatitis. No further biopsies were taken. No significant clinical improvement was observed post-treatment (Fig. 3c).

## Serological analyses

Banked serum from the two donkeys was submitted for serological analysis. The approach for serological analyses included ELISA (enzyme-linked immunosorbent assay) for screening, IFAT (immunofluorescence antibody test) for antibody titration and Western blot (WB) as the confirmatory technique. Antigens of *B. besnoiti* originally isolated from cattle were used, since strong cross reactions with the respective species infecting equids (*B. bennetti*) were previously demonstrated [29].

### ELISA

Serum samples were screened for *B. besnoiti* antibodies using a commercial ELISA kit (ID Screen® *Besnoitia* Indirect 2.0, Innovative Diagnostics, France) according to the instructions for use (IFU), with slight modifications. A detection antibody raised against equine immunoglobulins was used. Bovine positive and negative controls sera supplied in the kit, and positive and negative samples from equids [30], were included. Samples with sample/positive (S/P) ratios ≥ 30% were considered positive and submitted for IFAT and WB.

### IFAT

Sera were analysed for anti-*Besnoitia* spp. antibodies by IFAT, using slides coated with *B. besnoiti* antigens provided in a commercial kit (MegaFLUO® BESNOITIA besnoiti, Megacor, Austria), following the IFU, with slight modifications. An initial screening dilution of 1:50 was used according to Elsheikha et al. [23]; then, seropositive samples were two-fold serially diluted to determine the end-point antibody titer. A fluorescein isothiocyanate (FITC) anti-horse IgG was used as detection antibody (MegaFLUO® FITC anti-horse IgG Conjugate, Megacor, Austria). Positive and negative controls from bovine sera supplied in the kit, and positive and negative samples from equids [30], were also included in each assay. The slides were evaluated using a fluorescent microscope (Axioscope 2, Zeiss), comparing each well to the fluorescence of the positive and negative controls, considered for reference.

### Western blot

Confirmatory WB for *Besnoitia* spp. was performed and interpreted as previously described [30]. Pellets of *B. besnoiti* (Bb-Sp1) tachyzoites were purchased from Saluvet Innova (Spain): these antigens are obtained from cell cultures of the MARC-145 cell line, purified by gel filtration, washed with PBS containing 2 mM of protease inhibitor PMSF, and pelleted by centrifugation. A total of 4 × 10^7^ *B. besnoiti* tachyzoites resuspended with Laemmli buffer under non-reducing conditions were employed for electrophoresis. Electrophoresis was performed in 12% precast polyacrylamide gel (Mini-PROTEAN® TGX™ Precast Protein Gels, Bio-Rad Laboratories, USA) and then transferred to a nitrocellulose membrane (Mini Trans-Blot Cell, Bio-Rad Laboratories, USA). Precision Plus Protein™ Kaleidoscope™ Prestained Protein Standard (Bio-Rad Laboratories, USA) was included in the electrophoresis to estimate the apparent molecular weights of the different antigens recognized. Sera from the two donkeys were included at a 1:20 dilution, followed by a peroxidase-conjugated goat anti-donkey IgG (H + L) secondary antibody diluted at 1:1500 (Novus Biologicals Europe, United Kingdom). Positive and negative control sera, both from donkey [30] and cattle [31], were included. Cattle control sera were also tested at a 1:20 dilution, followed by a peroxidase-conjugated anti-Bovine IgG antibody diluted at 1:1200 (Sigma-Aldrich, USA). The presence of at least three bands in at least two of the three principal antigenic areas (area I: 72.5, 58.9, and 51.4 kDa; area II: 38.7, 31.8, and 28.5 kDa; area III: 23.6, 19.1, 17.4, 14.5 kDa) was considered a positive result for *Besnoitia* spp. [32].

**Fig. 5:**
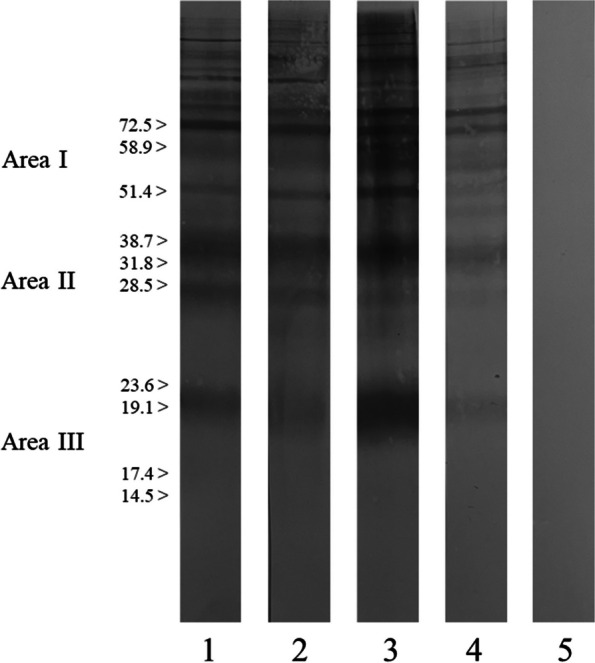
WB of *Besnoitia* spp. tachyzoite antigens probed with serum samples from the two Irish donkeys show typical positive molecular weight distribution patterns. 1: Case 1; 2: Case 2; 3: Donkey positive control; 4: Bovine positive control; 5: Negative control

Both donkeys tested positive by ELISA with S/P% values of 72.1 and 128.9 in cases 1 and 2, respectively. IFAT was positive in both cases with antibody titres of 1:400 and 1:1600, respectively. Both donkeys were confirmed positive to *Besnoitia* spp. specific antibodies by confirmatory WB (Fig. 5).

## Molecular analyses

Formalin fixed paraffin embedded (FFPE) tissues of skin from both cases were used for internal transcribed spacer 1 (ITS-1) PCR and microsatellite typing of *Besnoitia* spp.

### DNA extraction

For the PCR analysis 2 × 10 μm sections were taken aseptically from each FFPE block and placed in a 1 ml PCR Eppendorf tube in duplicate. DNA was extracted from these sections using a commercial kit (DNeasy Blood & Tissue Kit; Qiagen, Venlo, Netherlands). The pretreatment protocol for paraffin-embedded tissue as detailed in the IFU included deparaffinisation steps using xylene and ethanol.

The DNA concentrations were measured using the NanoDrop (Thermo Scientific™ NanoDrop) and samples were stored at -20ºC until analysis.

### ITS-1 PCR

DNA samples were initially subjected to real time PCR analysis with primers targeting the ITS-1 region as described by Cortes et al. [33] (Supplementary Table 1). Positive and negative (non-template) controls were included in each run; the positive control consisted of genomic DNA extracted from skin biopsy samples from a dairy cow chronically infected with *B. besnoiti* [31]. Each sample was analysed in duplicate, and the mean cycle threshold (Ct) and melting temperature (Tm) values were recorded.

Samples that tested positive by real time PCR were further analysed by conventional PCR and subsequent sequencing was carried out, with the same primers used for real time PCR, targeting a region of 231 bp of the ITS-1 region (Supplementary Table 1) [33]. The same positive and negative (non-template) controls described above were inserted in each run. PCR products were run on 1.5% agarose gel containing 0.05% ethidium bromide in TBE buffer electrophoresis and visualized under UV light on a transilluminator. Bands of the expected size were excised from agarose gel, purified with a commercial kit (NucleoSpin® Gel and PCR Clean-up, Macherey–Nagel, Düren, Germany) according to the IFU, and finally sent for bidirectional sequencing to a commercial service (Microsynth Seqlab GmbH, Germany). Electropherograms were checked, and consensus sequences were manually assembled. Sequences were compared to nucleotide sequences of *Besnoitia* spp. available in the GenBank using BLASTn (https://blast.ncbi.nlm.nih.gov/) and then aligned with sequences available in GenBank using the Mega6 software.

### Microsatellite typing

Samples positive for *Besnoitia* spp. DNA by both real time and conventional ITS-1 PCR, were subjected to microsatellite typing, using primers and protocols previously described [34], to amplify 6 microsatellite loci, i.e. Bt-5, Bt-6, Bt-7, Bt-9, Bt-20 and Bt-21 (Supplementary Table 2). The same positive and negative (non-template) controls described above for ITS-1 PCRs were included in each run. PCR products were run on 2% agarose gel containing 0.05% ethidium bromide in TBE buffer electrophoresis and visualized under UV light on a transilluminator. Bands of the expected size were excised from agarose gel, purified with a commercial kit (NucleoSpin® Gel and PCR Clean-up, Macherey–Nagel, Düren, Germany) following the IFU, and finally sent for bidirectional sequencing to a commercial service (Microsynth Seqlab GmbH, Germany). Electropherograms were checked, and consensus sequences were manually assembled. Sequences were compared to nucleotide sequences of each microsatellite locus for available *Besnoitia* spp. [34] and then aligned using the Mega6 software to count the number of repeat motifs for each marker.

Skin samples collected from both donkeys were positive for parasitic DNA by ITS-1 real time PCR. Mean quantification cycle (Cq) and Tm values were 25.9 and 81.0 in case 1 and 31.2 and 81.4 in case 2, respectively. Both real-time PCR positive samples were also confirmed by ITS-1 endpoint PCR. Sequencing of 231-bp PCR-fragments demonstrated no nucleotide variations between the two sequences (100% identity), and homology of 100% with *Besnoitia* spp. sequences deposited in GenBank. Of note, the presence of the T insertion at position 148 previously reported for *B. bennetti* was observed [24] (Supplementary Fig. 1). The obtained sequences were submitted to GenBank under accession numbers OR046541-OR046542.

Microsatellite typing showed the characteristic pattern of *B. bennetti* for both cases (Table 2). In locus Bt-7, nine repeats were found in the present study, which is similar to *B. bennetti* from the UK [23], and in contrast to *B. bennetti* from North American which shows eight repeats at locus BT-7 [3, 5, 21, 34] (Table 2).

**Table 2:** Comparison of six microsatellite loci of different *B. bennetti* isolates sequenced from infected donkeys

*Besnoitia* species	Host	Sample	Country	Microsatellite locus						Reference
				**Bt-5**	**Bt-6**	**Bt-7**	**Bt-9**	**Bt-20**	**Bt-21**	
*B. bennetti*	Donkey Case 1	Skin	Ireland	12	13	9	8	8	6	This study
	Donkey Case 2	Skin	Ireland	12	13	9	8	8	6	This study
	Donkey	Skin	UK	12	13	9	8	8	6	Elsheikha et al., 2020 [23]
	Donkey	Skin	Michigan, USA	12	13	8	8	8	6	Elsheikha et al., 2005 [21]; Madubata et al., 2012 [34]
	Donkey	Conjunctiva	Texas, USA	12	13	8	8	8	6	Ness et al., 2012 [5]; Gutierrez-Exposito et al., 2016 [3]

Based on the findings of the clinical, histopathology and serology examination together with the molecular analysis, a diagnosis of besnoitiosis caused by *B. bennetti* was made for the two cases.

## Discussion and conclusion

This is the first report of besnoitiosis due to *B. bennetti* in Ireland. Both cases were identified in young (2–2.5 years old), systemically healthy donkeys, and clinical, histopathological, serological, and molecular techniques were required to reach a definitive diagnosis. The origin of the infection in these Irish donkeys is difficult to determine, especially as the life cycle and route of transmission remain unclear for *Besnoitia* spp. in equids. Both donkeys were born on a farm in north Cork. Two outbreaks of bovine besnoitiosis have previously been reported in Ireland in both dairy [35] and beef cattle [36] in Co. Tipperary and Co. Cork respectively. Although the *Besnoitia* species was not identified at molecular level in these cases, it was assumed to be *B. besnoiti*. Thus, these outbreaks are unlikely linked other than by location as all occurred in the Southwest of Ireland within a 100 km radius.

The initial diagnosis was made as an incidental finding in the dermis adjacent to a fibroblastic sarcoid in case 1. Case 2 presented with numerous areas of non-pruritic, focal alopecia with crusts and nodules, some of which contained caseous exudate. While these findings are similar to clinical cases in British donkeys with besnoitiosis [23], the multifocal presence of calcinosis circumscripta has not been reported on the nose of donkeys before or in association with *Besnoitia* infection. This dystrophic mineralization occurs secondary to tissue injury and is likely the result of dying *Besnoitia* cysts. In case 2, calcinosis circumscripta may have contributed to the presence of larger dermal nodules, as compared to case 1, and to the caseous exudate seen. The presence of low numbers of large tissue cysts in host cells with thick collagenous outer walls has typically been described in besnoitiosis in donkeys [23, 24] and is characteristic of chronic infection [37, 38]. In both cases the size of the cysts varied considerably reaching up to 562 µm in length. No evidence of extracystic zoites was found but may have been missed in the absence of using immunohistochemistry [37].

Cysts were mainly present in the sclerae, conjunctivae, nares and muzzle in the two current cases, similar to previous reports where the sclera and the nares were the most common locations for *Besnoitia* lesions in donkeys [21, 25, 27]. No endoscopic examination was performed to look for the presence of laryngeal or nasopharyngeal cysts, which are less commonly identified [5], and vaginal mucosal cysts were not seen in the jenny here but had been identified in several cases previously [1, 5].

The impact of *Besnoitia* sp. infection on equine health and welfare remains debatable. The two young donkeys in this report were healthy, showed only mild dermal signs and had unremarkable blood results. This is similar to other studies, where *Besnoitia* cysts were noted incidentally at post-mortem examination in donkeys that were euthanized for other reasons [23, 28]. In contrast, single cases of equine besnoitiosis in South Africa [18], the USA [1, 21, 27], Belgium [24], and Italy [25] were reported as severely affected, demonstrating cachexia and/or debilitation. However, co-morbidities, such as significant burdens of endo- and ecto-parasites [25] were often found in these animals and were reflected in haematologic and biochemical alterations [1, 24, 25]. In the case of a South African pony [18], a link between cutaneous and laryngeal *Besnoitia* cyst manifestation and purulent nasal discharge, dyspnoea and stridor and hyperglobulinaemia on biochemistry, remained unclear as no further diagnostic testing was performed to investigate the respiratory signs further. Interestingly, haematologic and biochemical alterations have been reported in *B*. *besnoiti* infected cattle [39] but the observed changes were generally transient, mild and non-specific. In the authors’ opinion, haematology and serum biochemistry remain important tests in debilitated animals with weight loss and cachexia due to any cause, but care should be taken not to assign non-specific haematologic and biochemical alterations exclusively to a diagnosis of besnoitiosis.

One of the donkeys described here had *Besnoitia* cysts adjacent to a sarcoid in the dermis, which was associated with an old scar. Open wounds or areas of skin trauma are more likely to be affected by sarcoids [40] and may predispose to *Besnoitia* spp. infection. The intradermal route of infection by bradyzoites was shown to be more effective than other routes in cattle [41] and insects as mechanical vectors and direct contact between animals have been shown to play a role in the spread of the parasite infection [12, 13]. Transmission by direct contact between donkeys has also been reported [16]. The relationship between the presence of sarcoids and *Besnoitia* cysts in donkeys was previously observed in UK donkeys [23] whereby the cysts were found within and nearby sarcoids in 5/8 masses from donkeys submitted for histopathology and in 3/12 donkeys submitted for post-mortem examination. Whether this is an incidental finding due to the higher prevalence of sarcoids in donkeys, or if the presence of *Besnoitia* cysts or sarcoids allows for the establishment of the other is unclear [23]. There is an association between equine sarcoid susceptibility and genetic traits [42], and other genes related to immune response all playing an important role in sarcoid development [43], which may enhance susceptibility to *Besnoitia* infection. Based on the above findings, besnoitiosis should be considered as a differential diagnosis for cutaneous masses in donkeys with or without sarcoids, especially in younger donkeys.

It has been reported across studies, both in North America and in Europe, that besnoitiosis is more common and more severe in younger animals, 1–3 years of age, [1, 5, 19–21, 25]. However, the disease (cutaneous and ocular cysts) has also been described in older animals [1, 23, 24]. Stress has been identified as a possible risk factor for more severe disease in younger donkeys, that were exposed to stressful housing conditions or were recently transported [21].

Currently, there is no definitive effective treatment for the resolution and elimination of *Besnoitia* cysts in donkeys [1, 5, 20, 24]. Antibiotics with antiprotozoal activity (trimethoprim sulfadiazine or sulfamethoxazole) and two antiprotozoal drugs (ponazuril, nitazoxanide) have been used with variable doses and duration to treat besnoitiosis in donkeys [1, 5, 20, 24] but their efficacy is equivocal. In the two Irish donkeys described here cysts persisted after treatment with trimethoprim sulfadiazine twice daily for 30 days, however no repeat biopsies were performed. Spontaneous resolution of the disease has been reported in cattle [3] but has not been investigated in equids.

Both donkeys here were positive for *Besnoitia* spp. antibodies. For all the serological tests (ELISA, IFAT, and WB), antigens of *B. besnoiti* originally isolated from cattle were used, since strong cross-reactions with the respective species infecting equids, i.e. *B. bennetti*, have been demonstrated [29]. The use of Western blot as a confirmatory technique is highly recommended due to the possibility of cross-reactions between closely related Sarcocystidae [44]. IFAT results demonstrated a high antibody titre in both animals (1:400 and 1:1600 in donkey cases 1 and 2, respectively), suggesting active *Besnoitia* infection. However, current serological tests (ELISA, IFAT, WB) do not discern *Besnoitia* species, making *B. besnoiti* and *B. bennetti* infections serologically indistinguishable [45, 46]. Therefore, molecular methods are needed for the aetiological diagnosis of besnoitiosis. In our case report, microsatellite typing confirmed that the *Besnoitia* species detected in Irish donkeys was *B. bennetti*. Indeed, all the 6 microsatellite markers examined were identical to those reported in *B. bennetti* from the UK. The Irish isolates characterized here and, in the UK [23], were also identical to those from the USA. Only a single repeat more at the microsatellite locus Bt-7 was seen in the European *B. bennetti* isolates compared to the North American isolates [4, 5, 21, 34]. In addition, ribosomal DNA ITS1 sequences for *B. bennetti* of the two Irish cases had the same sequence with the same T insertion at position 148 as isolates from donkeys in the USA and Belgium [5, 24]. However, more DNA markers from diverse *B. bennetti* isolates are needed to enrich molecular databases and elucidate the parasite population genetic structure [23]. The genetic data would further help explain the epidemiology of this newly emerging pathogen in Europe.

The origin of the infection in this Irish herd was unclear. The two donkeys lived on the same premise in two separate groups, which were out on strip-grazed pasture between April and November and were then kept in yards for the autumn and winter. Apart from donkeys, mules, and one pony (approximately 500 equids on the farm), other animals present on this farm included grazing sheep a few months a year, three barn cats, and the occasional dog. Wild animals reportedly observed include rabbits, red fox, and rodents, some of which could be a potential definitive host. *Besnoitia* spp. DNA was detected recently in faecal samples from four red foxes (V*ulpes vulpes*) [47] in Spain. Serological results also suggested the role of African lions (Panthera leo) and wild cats (*Felis sylvestris catus*) in the biological cycle of the parasite [48, 49]; however, attempts to confirm domestic cats as the definitive host have been unsuccessful [1]. Trans-boundary animal trade and movement was identified as a risk factor for the establishment of new outbreaks of bovine besnoitiosis in naive areas [2, 50]. Interestingly, one of the clinically affected donkeys reported in the study from the UK [23] originated from the Irish herd of The Donkey Sanctuary. Direct contact between animals and insects as mechanical vectors has been suggested to play a role in transmission of *Besnoitia sp*. in donkeys [1, 5], but this has not been proven. Some herd mates of the two Irish donkeys in this report were found to have *Besnoitia* cysts and further examination and investigation is underway to assess the extent of the clinically affected animals in the herd and monitor disease progression on the farm.

This is the first report of *B. bennetti* in Ireland. It confirms the spread of equine besnoitiosis as an emerging disease in donkeys across Europe. Our study describes the clinical features, diagnoses and treatment in detail, to raise awareness among veterinary practitioners of this parasitic disease and its consequences on the health and welfare of equids, especially donkeys. Besnoitiosis should be included among differential diagnoses for skin lesions in donkeys, particularly in cases of cutaneous masses, non-pruritic dermatitis, and dermatitis that is not responsive to treatment. This study further highlights the role of histopathology in making the initial diagnosis and the importance of molecular analysis and genetic sequencing for exact species identification. Future studies should aim to get a better understanding of the epidemiology of *Besnoitia* infection in donkeys in relation to both serological prevalence, transmission within a herd and clinical impact. Further investigations into parasite biology, phylogeny, and transmission modes are necessary to understand this emerging pathogen.

### Supplementary Information

**Additional file 1: Supplementary Figure 1.** ITS1 sequence alignment for the Besnoitia spp. isolated from the two Irish donkeys show an insertion of T at position 148 characteristic for *B. bennetti*.

**Additional file 2: Supplementary Table 1. **ITS-1 real time and conventional PCR primers sequences, amplicon sizes and conditions. **Supplementary Table 2.** Microsatellite loci for *Besnoitia *spp. typing: names, primers and repeat sequences, amplicon sizes and PCR conditions.

## Data Availability

All data are presented within the paper.

## Abbreviations

B.: Besnoitia
BID: Two times a day
bpm: Beats per minute
cm2: Square centimetre
Cq: Quantification cycle
DNA: Deoxyribonucleic acid
dL: Decilitre
ELISA: Enzyme-linked immunosorbent assay
FITC: Fluorescein isothiocyanate
g: Gram
IFAT: Immunofluorescence antibody test
IFU: Instructions for use
ITS: Internal transcribed spacer
IgG: Immunoglobulin G
kDa: Kilodalton
kg: Kilogram
mg: Milligram
mm: Millimetre
PCR: Polymerase Chain Reaction
PO: Per os
RR: Reference range
S/P: Sample/positive
spp.: Species
TBE: Tris/Borate/EDTA
Tm: Melting temperature
UCD: University College Dublin
UK: United Kingdom
USA: United States of America
UV: Ultraviolet
WB: Western blot
µM: Micrometre
