# Supplementary material for: First report of Besnoitia bennetti in Irish donkeys: an emerging parasitic disease in Europe
Source: Ir Vet J. 2024 Feb 14;77:2. doi: 10.1186/s13620-024-00263-2 (PMC10865628; doi:10.1186/s13620-024-00263-2)
Supplement: Supplementary file 1 — Additional file 1: Supplementary Figure 1. ITS1 sequence alignment for the Besnoitia spp. isolated from the two Irish donkeys show an insertion of T at position 148 characteristic for B. bennetti. [file 13620_2024_263_MOESM1_ESM.rtf]

Alignement of ITS1


                             10         20         30         40         50         60         70         80         90        100                           
                      
MG652473 B. bennetti  TGTCCTTTTG ACATTTAATA ACAATCAACC CTTGAATCCC TATTACAACA ATAAGCTTGC ATCTCTCGTT TCGAGGGGTG CATTCGAGAA GTGTGCTGCC 
JQ013812 B. bennetti  .......... .......... .......... .......... .......... .......... .......... .......... .......... ..........  
AY665399 B. bennetti  .......... .......... .......... .......... .......... .......... .......... .......... .......... ..........  
JF314861 B. besnoiti  .......... .......... .......... .......... .......... .......... .......... .......... .......... ..........  
HM008988 B. caprae    .......... .......... .......... .......... .......... .......... .......... .......... .......... ..........  
AY665400 B. tarandi   .......... .......... .......... .......... .......... .......... .......... .......... .......... ..........  
Donkey Case 1         .......... .......... .......... .......... .......... .......... .......... .......... .......... ..........  
Donkey Case 2         .......... .......... .......... .......... .......... .......... .......... .......... .......... ..........  

                            110        120        130        140        150        160        170        180        190        200                  

MG652473 B. bennetti   CTCTTGTTGT CATTTTTGAC AACAAGAGCA TCGCCTTCTT  TTTTTTTTCC  AACACCGTTT AACTAAACCA ACGATCTGTT GTTTAGCGGG CGGGGATCCT 
JQ013812 B. bennetti  .......... .......... .......... .......... ........T.. .......... .......... .......... .......... .......... 
AY665399 B. bennetti  .......... .......... .......... .......... ........T.. .......... .......... .......... .......... ..........  
JF314861 B. besnoiti  .......... .......... .......... .......... ........-.. .......... .......... .......... .......... .......... HM008988 B. caprae    .......... .......... .......... .......... ........-.. .......... .......... .......... .......... .......... AY665400 B. tarandi   .......... .......... .......... .......... ........-.. .......... .......... .......... .......... .......... 
Donkey Case 1         .......... .......... .......... .......... ........T.. .......... .......... .......... .......... .......... 
Donkey Case 2         .......... .......... .......... .......... ........T.. .......... .......... .......... .......... .......... 

                             210        220        230   	  240  
                      
MG652473 B. bennetti  CACCTCCTCA CTGCTATCAC GGATTGGTTA ATACAAACCT TTTG   
JQ013812 B. bennetti  .......... .......... .......... .......... ....
AY665399 B. bennetti  .......... .......... .......... .......... ....
JF314861 B. besnoiti  .......... .......... .......... .......... ....
HM008988 B. caprae    .......... .......... .......... .......... ....
AY665400 B. tarandi   .......... .......... .......... .......... ....
Donkey Case 1		  .......... .......... .......... .......... ....
Donkey Case 2     	  .......... .......... .......... .......... ....  
