# Supplementary material for: First report of Besnoitia bennetti in Irish donkeys: an emerging parasitic disease in Europe
Source: Ir Vet J. 2024 Feb 14;77:2. doi: 10.1186/s13620-024-00263-2 (PMC10865628; doi:10.1186/s13620-024-00263-2)
Supplement: Supplementary file 2 — Additional file 2: Supplementary Table 1. ITS-1 real time and conventional PCR primers sequences, amplicon sizes and conditions. Supplementary Table 2. Microsatellite loci for Besnoitia spp. typing: names, primers and repeat sequences, amplicon sizes and PCR conditions. [file 13620_2024_263_MOESM2_ESM.docx]

**Supplementary Table 1:** ITS-1 real time and conventional PCR primers sequences, amplicon sizes and conditions.

| **Method** | **Target gene** | **Primers (5’ → 3’)** | **Amplicon size** | **Conditions** |
| --- | --- | --- | --- | --- |
| Real time PCR | ITS-1 | F: GACATTTAATAACAATCAACCCTT  R: GGTTTGTATTAACCAATCCGTGA | 231 bp | Incubation at 50 °C for 2 min, denaturation at 95 °C for 2 min, amplification for 40 cycles at 95 °C for 15 s, 60 °C for 60 s, and a final melting analysis step. |
| Conventional PCR | ITS-1 | F: GACATTTAATAACAATCAACCCTT  R: GGTTTGTATTAACCAATCCGTGA | 231 bp | Initial denaturation at 95 °C for 3 min, 40 cycles of denaturation (30 s at 95 °C), annealing (45 s at 58 °C) and extension (1 min at 72 °C), and a final extension step at 72 °C for 10 min. |

**Supplementary Table 2:** Microsatellite loci for *Besnoitia* spp. typing: names, primers and repeat sequences, amplicon sizes and PCR conditions.

| **Bt** | **Primers (5’ → 3’)** | **Repeat sequence** | **Amplicon size** | **Conditions** |
| --- | --- | --- | --- | --- |
| Bt-5 | F: AGAGACTCAATGAACCAAGC  R: GTTATTCTCCTTTCCCCTTC | ACACACACACACACACACACACAC | 235 bp | Initial denaturation at 95 °C for 3 min, 40 cycles of denaturation (30 s at 95 °C), annealing (1 min at 53 °C for Bt-5, Bt-6, Bt-7, Bt-9, Bt-21 or at 51°C for Bt-20) and extension (1,5 min at 72 °C), and a final extension step at 72 °C for 10 min. |
| Bt-6 | F: GAGGTAGTCAGATTGGAACG  R: GTCAATGAACTCGTGGATCT | ACACACACACACACACACACACACAC | 241 bp |  |
| Bt-7 | F: GTTTTCCTTTCCACATTCG  R: GAGCAGAGAACGAGAAGCTA | ACACACACACACACACAC | 151 bp |  |
| Bt-9 | F: GCTTTTATGATTGCTGGAAC  R: GTTGAATACGCGATCAATTT | ACACACACACACACAC | 157 bp |  |
| Bt-20 | F: ACAAGAATTTGTGGATACCG  R: TCTAAATCGAGGCCATACCAT | CACACACACACACACA | 232 bp |  |
| Bt-21 | F: GTGAAAGCTGCGATATAACC  R: ACACATTCGCCTTGGTTT | CACACACACACA | 122 bp |  |
